# Healthcare Decision-Making Ecosystem Deengineering

**DOI:** 10.7759/cureus.97719

**Published:** 2025-11-25

**Authors:** Stylianos Papalexandris, Thomas Fotiadis, Lamprini Seremeti, Dimitrios Folinas, Sofia D Anastasiadou

**Affiliations:** 1 Department of Production and Management Engineering, Democritus University of Thrace, Xanthi, GRC; 2 Department of Agricultural Economics and Rural Development, Agricultural University of Athens, Amfissa, GRC; 3 Department of Supply Chain Management, International Hellenic University, Katerini, GRC; 4 Department of Midwifery, University of Western Macedonia, Ptolemaida, GRC; 5 School of Economics, Administration and Computer Science, University Neapolis Pafos, Pafos, CYP

**Keywords:** decision-making, deengineering, ecosystem, healthcare, leadership, organisational culture

## Abstract

Decision-making in healthcare is not just a singular, straightforward process but rather a complex and continuously evolving interplay of heterogeneous interconnected factors. Decision theories and reasoning methods, stakeholders’ inherent qualities, leadership models, diversity in organization culture, regulatory frameworks, and technological advancements all contribute to shaping the modern healthcare decision-making ecosystem. While each of these constituent elements intends to enhance the robustness and quality of decisions, their implicit influences often introduce additional layers of complexity. This article uses the notion of de-engineering in order to introduce an approach for setting apart and critically examining the individual components of the healthcare decision-making ecosystem. By identifying the strengths and limitations of the constituent elements, the de-engineering approach serves as a foundational step toward redesigning more resilient and effective healthcare decision-making.

## Introduction and background

Decision-making is an essential aspect of medical practice [1] and a vital component of the modern healthcare ecosystem [2-3]. From choosing the right treatment [4] to allocating resources to intensive care units [5], the decisions made every day in hospitals, primary care, and public agencies directly affect patients' lives, the effectiveness of services, and the sustainability of the health sector [6]. However, the complexity of modern medical practice, combined with the abundance of data [7], fast-moving technologies [8], and unstable regulatory frameworks [9], makes the integrated decision-making healthcare system a complex and multi-layered entity [10]. By “unstable regulatory framework,” we refer to the continuously evolving healthcare policies developed, introduced and applied by key stakeholders to address emerging challenges. From this perspective, the political landscape and its influence may drive organizational adaptation, especially within the public sector, where policies often need to align with prevailing political directions.

Traditionally, medical decisions were based primarily on the experience and judgment of the clinician [11]. The rapid digitization of health information [12], the diffusion of decision support tools (Clinical Decision Support Systems - CDSS) [13], and the rise of institutional, legal, and ethical constraints [14] have transformed the healthcare decision-making ecosystem [15]. Nowadays, decisions are not the result of individual clinical actions, but the product of collective, interoperable, and sometimes algorithmically assisted processes. Indeed, more and more active or passive parts, whether technological systems (e.g., CDSS, AI-based systems) or organizational entities (e.g., protocols, committees), are embedded into everyday clinical practice in the name of optimization [16]. However, the complexity of their interplay often obscures the critical elements that lead to the final choice. Decision thresholds and criteria may be opaque [17], arbitrary, or inconsistent with patient needs and ethical principles of care [18].

In this context, de-engineering of the healthcare decision-making ecosystem is proposed as a methodological tool for understanding its mechanisms and overall behaviour, by critically evaluating existing limitations and strengths of its constituent elements. While engineering implies designing and building complex, large-scale systems, de-engineering conceptually means the opposite, that is, taking apart a system to return it to a less complex state or to its fundamental elements [19-20]. In this approach, de-engineering is understood as the analytical deconstruction of a coherent system into its basic subsystems, with the aim of (a) highlighting its constituent elements and the relationships between them, (b) revealing their explicit or implicit strengths and limitations, and (c) prompting the challenges of reconstructing the decision under more transparent and reflective conditions. This paper adopts a conceptual design, since it aims to articulate and justify a de-engineering perspective on healthcare decision-making by integrating established theories and domains concerning decision theory, stakeholder roles, leadership, culture, regulation, and emerging technologies into a coherent analytic frame. In this vein, it synthesizes diverse literatures to clarify mechanisms, expose hidden couplings, and propose an operational vocabulary and steps that can guide future empirical and design work.

The remainder of this paper is organized into two key sections. First, it explores the foundational elements of the healthcare decision-making ecosystem, including decision theories and reasoning models, stakeholders’ inherent qualities and roles, leadership paradigms, organizational cultures, regulatory frameworks, and emerging technologies. The second section examines the dynamic interplay among these elements and how their interactions contribute to the complexity and unpredictability of decision-making in healthcare settings. Finally, the paper concludes by offering recommendations for building more resilient, adaptive, and transparent healthcare decision-making frameworks. By presenting a holistic de-engineering approach, this paper seeks to illuminate hidden dependencies, challenge conventional assumptions, and provide a structured pathway for critically reassessing and strengthening healthcare decision-making systems.

## Review

Constituent elements of the healthcare decision-making ecosystem

This section broadly surveys the basic building blocks of the healthcare decision-making ecosystem, that is, decision theories and reasoning models, stakeholders’ inherent qualities and roles, leadership paradigms, organizational cultures, regulatory frameworks, and emerging technologies. It conceptualizes these core components in order to map out their aims, strengths, and limitations, and to pinpoint the decision points where they exert the greatest influence. Given that the paper is conceptual, the aim here is to map the healthcare decision-making ecosystem in simple terms. The literature was gathered through a purposive, structured search of major scholarly databases, selecting English-language works published primarily between 2015 and 2025 that directly address decision-making or one of its core components (decision theories and reasoning, stakeholder roles, leadership paradigms, organizational culture, regulation, and emerging technologies). Opinion pieces without methodological grounding, sources outside health with limited transferability, and materials lacking sufficient detail were excluded. The synthesis was thematic and conceptual with a narrative presentation; that is, findings were first grouped by core element, and then the cross-cutting interactions among them were highlighted.

Decision Theories and Reasoning Models

Healthcare decision-making involves multiple theoretical frameworks, such as decision-making theories and reasoning models, which help explain how stakeholders make choices in clinical contexts.

Decision-making theories provide structured frameworks for understanding how decisions are made, evaluated, and improved [21]. In healthcare, these theories support both clinical judgments and administrative choices, offering models that guide decision-makers in situations of uncertainty, complexity, and competing values [11]. In general, decision-making theories can be categorized into three main types: normative, descriptive, and prescriptive [22]. Within this classification, basic models such as the Rational Model, Bounded Rationality, Health Belief Model, and Intuitive Decision-Making offer specific perspectives on how decisions unfold in practice.

Normative decision theory outlines how decisions should be made to achieve optimal outcomes [23]. Rooted in logic, mathematics, and probability, the Rational Model is a foundational normative approach [24]. It assumes decision-makers have access to complete information, clearly defined goals, and the cognitive capacity to evaluate all options before selecting the one that maximizes utility. In healthcare, this model informs clinical guidelines, treatment algorithms, and resource allocation frameworks [25]. However, its assumptions rarely hold in real-world clinical environments characterized by time pressure, uncertainty, and incomplete data.

Descriptive decision theory investigates how decisions are made in reality, often diverging from rational ideals due to human cognitive and emotional limitations [26]. The Health Belief Model is a psychological framework used to explain and predict individual health behaviours by focusing on personal beliefs and perceptions [27]. Bounded Rationality recognizes that decision-makers operate under constraints such as limited time, information, and mental processing capacity [28]. Instead of optimizing, individuals often “satisfice”, that is, choose an option that is “good enough” rather than optimal [29]. Intuitive Decision-Making, another key descriptive model, emphasizes the role of experience, pattern recognition, and subconscious processing [30], particularly in high-stakes, time-sensitive settings like emergency medicine. While sometimes dismissed as subjective, intuitive judgments are often accurate, especially among seasoned clinicians [31].

Prescriptive decision theory seeks to bridge normative ideals and descriptive realities by designing methods to improve decision quality while accommodating human limitations [32]. Approaches like Shared Decision Making are prescriptive tools that incorporate elements of both rational analysis and user-centred design [33]. They explain how patients and clinicians negotiate choices [34]. These models aim to enhance decision outcomes without assuming perfect rationality or ignoring the heuristics and emotions that influence real-world behaviour [35].

Beyond decision theory, the process of reasoning plays a critical role in shaping how decisions are formed, interpreted, and acted upon in healthcare [36]. Different philosophical and cognitive approaches to reasoning influence both the structure of clinical judgment and the dynamics of administrative planning [37]. Among the most prominent are the rationalist, phenomenological, and hypothetico-deductive approaches, each offering distinct strengths and limitations in how healthcare problems are perceived and resolved.

Rationalist approaches are grounded in logic, general principles, and deductive reasoning. They assume that knowledge can be derived from abstract reasoning and theoretical constructs, often independent of direct experience [38]. In healthcare, rationalist reasoning supports the development and application of standardized protocols, clinical guidelines, and evidence-based practices [39]. This approach aligns closely with normative decision-making, where decisions are seen as logical conclusions drawn from established rules and empirical data.

Phenomenological approaches, by contrast, emphasize lived experience, subjectivity, and the context-specific meaning of health and illness [40]. Rooted in continental philosophy, phenomenology seeks to understand how individuals perceive and make sense of their conditions, experiences, and choices. In healthcare, this method is particularly valuable in patient-centred care, qualitative research, and narrative medicine, where understanding the patient's personal story is as critical as interpreting their clinical indicators [41]. Phenomenological reasoning complements descriptive decision-making by incorporating emotional, cultural, and existential dimensions that often defy reduction to purely rational models.

Hypothetico-deductive reasoning is a structured approach commonly used in clinical diagnostics [42]. It involves generating hypotheses based on initial data, testing those hypotheses through further inquiry or diagnostic tools, and refining or rejecting them based on evidence. This method combines inductive and deductive elements and is a core component of clinical decision-making under uncertainty. It supports prescriptive practices by integrating evolving information and guiding action through a process of iterative refinement [43].

Each of these reasoning methodologies contributes to a more holistic understanding of healthcare decision-making. Rationalist thinking provides structure and consistency; phenomenological reasoning ensures empathy and context; and hypothetico-deductive methods allow for adaptive, evidence-driven clinical problem-solving. Recognizing when and how to employ each approach is essential for navigating the complexity of the healthcare decision-making ecosystem.

Stakeholders’ Inherent Qualities and Roles

Healthcare decision-making is a multidimensional process shaped not only by systems, policies, and data but by the people who engage in and are affected by those decisions. Stakeholders in healthcare, ranging from clinicians and patients to administrators, policymakers, insurers, and technology developers, each bring distinct perspectives, responsibilities, and inherent qualities that influence how decisions are made, justified, and implemented [44]. Understanding the roles and characteristics of these stakeholders is essential to decode the complexity of the decision-making ecosystem [45] and identify opportunities for reform through approaches like de-engineering.

At the core of this ecosystem are clinicians, whose decisions are often the most immediate and impactful for patient outcomes. Physicians, nurses, pharmacists, and allied health professionals draw on a combination of scientific knowledge, clinical experience, and ethical judgment. Their decision-making is guided by professional standards, patient needs, and contextual pressures such as time constraints and organizational priorities. Inherent qualities such as clinical intuition, tolerance for ambiguity, and ethical commitment shape their approach to care. However, clinicians may also experience cognitive overload, heuristic biases, or moral distress, all of which can influence the quality and consistency of decisions [46].

Patients are also increasingly recognized as central stakeholders in decision-making, particularly within models such as Shared Decision Making (SDM) [47]. They contribute not only personal preferences and values but also emotional, cultural, and experiential knowledge that cannot be captured by clinical data alone. Inherent qualities such as health literacy, trust, risk perception, and prior experiences with the healthcare ecosystem profoundly affect patients' willingness and ability to participate in decisions [48]. Disparities in socioeconomic status, education, and access can further complicate patient engagement, highlighting the need for inclusive and empathetic decision-making frameworks.

In addition, administrators and healthcare managers play critical roles in shaping organizational decisions related to resource allocation, staffing, infrastructure, and policy implementation. Their decisions are typically driven by considerations of efficiency, safety, financial sustainability, and regulatory compliance. Inherent traits such as strategic thinking, systems awareness, and leadership style directly impact how decisions are communicated and operationalized across the institution [49]. Yet, administrative decision-making often occurs at a remove from frontline realities, which can lead to misalignment between policy and practice. 

Moreover, policymakers and regulators influence the broader framework within which healthcare decisions occur. Through legislation, funding models, accreditation standards, and public health initiatives, they shape the environment in which clinical and organizational decisions are made. These stakeholders often operate with a population-level perspective, guided by principles of equity, cost-effectiveness, and political accountability. However, inherent qualities such as political ideology, lobbying influence, and risk tolerance can affect the objectivity and responsiveness of policy decisions. 

Insurers and payers, including both public and private entities, determine what services are reimbursable, influencing both provider behaviour and patient access to care. Their decision-making is guided by cost-benefit analyses, actual data, and utilization trends [50]. While their inherent role is to manage financial risk and sustainability, they may inadvertently constrain clinical autonomy or patient choice, particularly when reimbursement structures conflict with individualized care needs. 

Technology developers and data scientists are emerging as increasingly influential stakeholders, particularly with the rise of digital health tools, artificial intelligence, and clinical decision support systems. These actors shape the tools that clinicians and administrators rely on for decision-making [51]. Inherent qualities such as innovation orientation, technical expertise, and commercial incentives can lead to advancements in decision quality, but also introduce risks related to algorithmic bias, data privacy, and user disengagement if not carefully aligned with clinical realities and ethical standards.

The interplay among these stakeholders introduces both synergies and tensions [52]. For instance, while clinician-patient collaboration is central to shared decision-making, it can be undermined by administrative or policy constraints. Similarly, efforts to implement data-driven tools may clash with the intuitive reasoning used by experienced practitioners. These interactions highlight the layered complexity of the decision-making ecosystem, where decisions are rarely isolated and often involve negotiation, compromise, and multi-level coordination.

Critically, the inherent qualities of stakeholders, ranging from cognitive styles and ethical orientations to institutional power and communication skills, can amplify or inhibit the effectiveness of decision-making. Understanding these attributes is a necessary first step in any attempt to de-engineer the healthcare ecosystem. By dissecting not just stakeholder roles but also their dispositions, limitations, and biases, a clearer insight can be gained into how decisions are shaped and how they might be improved through system redesign.

In conclusion, stakeholders are not passive components in the healthcare decision-making ecosystem. They are active agents whose qualities and interactions fundamentally shape decision processes and outcomes. Any effort to improve or re-engineer healthcare decision-making must begin with a critical examination of who these stakeholders are, what drives their decisions, and how their diverse roles intersect.

Leadership Paradigms

Leadership plays a critical role in shaping how decisions are made, communicated, and implemented across the healthcare ecosystem [53-55]. In environments characterized by high complexity, resource constraints, ethical dilemmas, and rapid technological change, effective leadership is not merely about authority or control. It is about guiding collective decision-making toward outcomes that are patient-centred, evidence-informed, and operationally sustainable [56]. The leadership paradigm adopted within an organization directly influences the structure and inclusivity of decision-making processes [57-59]. Understanding different leadership styles and how they support or hinder effective decision-making is essential to dissecting and reimagining the healthcare decision-making ecosystem. Across leadership paradigms, Emotional Intelligence (EI) operates as a cross-cutting capability that fosters psychological safety, high-quality communication, and sense-making under uncertainty. Consequently, EI may shape the type of leadership applied and enable the translation of organizational values into day-to-day decision practices and strengthen the reliability of team-based outcomes.

Among the most widely studied and applied models in healthcare are transformational, transactional, servant, autocratic, and distributed leadership styles [60]. Each paradigm reflects a unique set of assumptions about human motivation, organizational behaviour, and the role of leaders in shaping outcomes [61].

Transformational leadership is one of the most prominent and often idealized paradigms in modern healthcare. Transformational leaders seek to inspire and motivate their teams by articulating a compelling vision, fostering innovation, and aligning individual values with organizational goals. This leadership style is associated with higher levels of team engagement, satisfaction, and adaptability, factors that are especially important in high-stakes decision-making environments such as hospitals and emergency departments. By promoting a culture of trust and empowerment, transformational leadership can facilitate inclusive decision-making, encourage shared ownership of problems, and support organizational change initiatives [62-63]. 

In contrast, transactional leadership is built on a foundation of clearly defined roles, expectations, and performance-based rewards or penalties. It is often more effective in highly regulated or procedural environments, such as in clinical compliance or administrative operations. While it may lack the inspirational qualities of transformational leadership, transactional leadership provides structure and consistency, attributes that are critical for ensuring accountability and operational efficiency. In decision-making, this style emphasizes adherence to protocols and standard operating procedures, which can enhance reliability but may suppress creativity or team input in complex, ambiguous situations [64]. 

Servant leadership focuses on meeting the needs of others first, particularly team members and patients. This paradigm prioritizes empathy, listening, stewardship, and the development of people, making it highly compatible with patient-centred care models. In the context of decision-making, servant leadership promotes a collaborative, bottom-up approach that values the perspectives of frontline staff and patients. By creating psychologically safe environments, servant leaders enable open dialogue and shared responsibility, particularly useful when navigating ethical dilemmas or care planning involving vulnerable populations [65]. 

Autocratic leadership, though increasingly out of favour in modern healthcare, still appears in high-pressure or hierarchical environments, particularly where decisions must be made quickly or where regulatory requirements leave little room for flexibility. This style centralizes authority and minimizes input from subordinates. While it can deliver fast decisions in crises, it often limits engagement, reduces transparency, and may lead to decision fatigue or resistance among team members [66]. 

A more contemporary approach is distributed or shared leadership, which views leadership as a collective function distributed across different roles and organizational levels. In complex healthcare settings where no single individual can possess all the necessary expertise, distributed leadership allows for more flexible, cross-functional decision-making. This paradigm supports collaborative models such as shared governance in nursing or multidisciplinary team-based care, where diverse expertise contributes to holistic decision-making [67].

Importantly, leadership paradigms do not operate in isolation [68]. They interact with organizational culture, stakeholder dynamics, and systemic constraints. For instance, a transformational leader in a rigid bureaucratic environment may find it difficult to implement change, just as a distributed leadership model may flounder in the absence of clear communication structures or accountability mechanisms. Additionally, external factors such as regulatory pressures, financial incentives, and patient expectations can influence how leadership is enacted in practice.

Leadership paradigms also have implications for equity and inclusion in decision-making [69]. For example, autocratic or transactional styles may inadvertently exclude marginalized voices, while servant and distributed models may help democratize decision processes and promote cultural competence. As such, leadership style is not just a managerial consideration but an ethical and strategic variable in healthcare decision-making.

In conclusion, leadership is a central mechanism through which healthcare decisions are shaped and executed. Each leadership paradigm brings distinct strengths and trade-offs, influencing not only the outcomes of individual decisions but also the broader culture and adaptability of healthcare organizations. As part of a de-engineering approach, critically examining leadership styles can reveal hidden power structures, value assumptions, and operational barriers that affect decision quality. By aligning leadership approaches with the goals of collaborative, transparent, and resilient decision-making, the healthcare ecosystem can move closer to delivering truly integrated and patient-centred care.

Organizational Cultures

Organizational culture plays a pivotal role in shaping how decisions are conceived, communicated, and enacted within the healthcare ecosystem [70]. It serves as the invisible yet powerful framework of shared values, norms, beliefs, and behaviours that influence how individuals and groups interpret information, assess risks, resolve conflicts, and allocate responsibility [71]. In the healthcare decision-making ecosystem, organizational culture affects not only clinical care but also leadership styles, communication patterns, staff engagement, and ethical values. As such, it is a foundational element that must be understood, critiqued, and, where necessary, reconfigured, particularly in the context of de-engineering complex decision-making ecosystems.

Healthcare organizations, whether hospitals, clinics, or public health institutions, often operate within multilayered cultures that reflect professional subcultures (e.g., nursing vs. medical), organizational histories, and broader systemic influences [72]. These cultural patterns manifest in decision-making through attitudes toward authority, openness to innovation, risk tolerance, collaboration, and accountability [73].

Among widely used Schneider’s, Trompenaars’, and Quinn and McGrath’s cultural typologies, Handy’s framework is especially adopted due to its clarity, applicability to healthcare organizations, and its emphasis on structural and power dynamics that directly influence decision-making processes [74]. It includes power culture, role culture, task culture, and person culture. In many healthcare settings, a role culture dominates, emphasizing defined hierarchies, standard procedures, and accountability through formal roles and rules. While this can support consistency and legal compliance, it may also stifle innovation and discourage front-line input, particularly in dynamic or complex care scenarios. In contrast, task cultures, often seen in high-functioning surgical teams or research units, prioritize problem-solving, collaboration, and results. These environments typically foster multidisciplinary decision-making and empower individuals based on expertise rather than rank. Such cultures are well-suited to complex, adaptive decision-making but may be difficult to sustain without strong leadership and clear communication channels. Power cultures, characterized by centralized control and top-down decision-making, are common in institutions where authority is concentrated among senior clinicians or executives. While they may allow for rapid decision-making in crisis situations, they often limit transparency and exclude non-dominant voices, such as nurses, allied health professionals, or patients. This can lead to decisions that are technically sound but lack contextual sensitivity or ethical balance. Person cultures, which place individual autonomy at the centre, are rare in institutional healthcare but may appear in patient-centred care models or in loosely affiliated networks such as professional associations. In such settings, decision-making tends to be highly individualized, which can enhance personalization but may also lead to fragmentation and inconsistency.

Importantly, organizational culture also influences how errors and uncertainty are managed, two fundamental aspects of healthcare decision-making. In a blame culture, errors are met with punishment and secrecy, discouraging open discussion and learning [75]. In contrast, a just culture promotes accountability while recognizing that human error is inevitable and often systemic. Organizations with a just culture are more likely to make transparent, data-informed decisions that prioritize patient safety and continuous improvement [76].

Culture also shapes communication and interprofessional collaboration, both of which are critical to complex decision-making [77]. Hierarchical cultures may inhibit junior staff from questioning senior clinicians, even when patient safety is at risk [78]. In contrast, cultures that foster psychological safety encourage open dialogue, feedback, and dissent, all of which contribute to more robust and inclusive decisions.

Moreover, organizational culture is not static [79]. It evolves in response to external pressures such as regulatory changes, financial constraints, patient expectations, and technological disruption. For instance, the introduction of electronic health records (EHRs), artificial intelligence, or population health mandates can shift decision-making cultures by introducing new values, such as data-driven accountability or algorithmic authority, into traditional clinician-led processes.

In the context of de-engineering, organizational culture must be examined not as a background variable but as an active force that enables or constrains decision quality. Dissecting culture involves identifying informal power dynamics, unwritten rules, and shared assumptions that may reinforce inefficiencies, inequities, or resistance to change. For example, a culture that rewards individual performance over team success may unintentionally discourage collaboration, while one that values tradition over evidence may resist innovation, even when patient outcomes are at stake.

In conclusion, organizational culture is a foundational yet often underexamined element of the healthcare decision-making ecosystem. It shapes not only what decisions are made but how, by whom, and to what end. By critically analyzing cultural patterns and their influence on decision-making, healthcare leaders, policymakers, and scholars can better identify misalignments between values and practices and begin to reconfigure cultures that support transparency, inclusivity, adaptability, and ethical care. Within the broader framework of de-engineering, organizational culture serves as both a site of constraint and a lever for transformation.

Regulatory Frameworks

Regulatory frameworks form the structural backbone of healthcare systems, setting the legal, ethical, and procedural boundaries within which decisions are made. In particular, with the integration of innovative tools in healthcare practice, new regulations have been developed [80-81]. These frameworks are designed to ensure patient safety, promote equity, standardize quality of care, and uphold public accountability [82]. However, while regulation provides necessary safeguards and guidance, it also introduces layers of complexity that shape and sometimes constrain the healthcare decision-making ecosystem. Understanding how regulatory frameworks influence decisions at both the clinical and administrative levels is essential for any effort to de-engineer and reimagine more responsive, resilient, and equitable decision-making.

Healthcare regulation operates across multiple levels: international, national, regional, and institutional. At each level, a diverse array of actors, including government agencies, professional licensing bodies, accreditation organizations, and ethical review boards, exert influence over decision-making processes. These regulatory bodies create and enforce policies that dictate standards of care, data privacy, reimbursement models, licensing requirements, reporting obligations, and more [83]. In this vein, de-engineering proceeds differently across jurisdictions. In more centralized regimes, it focuses on simplifying compliance pathways and clarifying decision rights within dense rule sets, whereas in more decentralized regimes, it emphasizes stitching together fragmented incentives and documenting minimum common thresholds to ensure transparency and fairness.

At the clinical level, regulations shape decisions through evidence-based practice guidelines, clinical protocols, informed consent procedures, and scope-of-practice rules. These frameworks aim to protect patient safety and reduce variability in care by anchoring decisions in standardized, peer-reviewed knowledge. For instance, decisions around prescribing medications or ordering diagnostic tests must align with national formulas, clinical pathways, and licensing restrictions. While such guidelines enhance consistency and legal defensibility, they can also restrict clinical autonomy, especially in complex cases where rigid adherence to protocol may not serve the patient's best interest. At the administrative and organizational levels, regulatory frameworks influence decision-making through accreditation standards, financial compliance requirements, labour laws, and public health mandates. Healthcare administrators must navigate a complex environment of healthcare financing regulations, data protection laws (e.g., GDPR), and safety accreditation systems (e.g., ISO). These rules affect decisions related to staffing, budgeting, infrastructure investment, and health information technology. Compliance is not optional; failure to meet regulatory standards can result in legal penalties, loss of accreditation, or reputational damage. At the individual level, ethical regulation, such as that provided by institutional review boards or clinical ethics committees, also plays a critical role in healthcare decision-making [14]. These bodies provide oversight for decisions involving vulnerable populations, experimental treatments, or end-of-life care. Their frameworks ensure that healthcare decisions respect human dignity, autonomy, and justice, but they can also slow down time-sensitive decisions or introduce additional bureaucratic layers.

While regulatory frameworks are intended to enhance accountability and protect public interest, they can also introduce unintended consequences. Excessive regulation may lead to defensive medicine, where clinicians make decisions primarily to avoid liability rather than optimize outcomes [84]. Administrative burdens associated with documentation, audits, and compliance reporting can divert attention and resources away from patient care [85]. Moreover, rigid or outdated regulations can stifle innovation, particularly when emerging technologies or personalized medicine approaches outpace existing legal and ethical guidelines [86].

From a holistic perspective, regulatory frameworks interact dynamically with other elements of the healthcare decision-making ecosystem, such as leadership styles, organizational culture, and stakeholder behaviour. For instance, a highly risk-averse regulatory environment may foster autocratic leadership and suppress frontline innovation, while a more flexible, principle-based regulatory model may support distributed leadership and adaptive problem-solving.

Within a de-engineering approach, regulatory frameworks must be critically examined not only for their explicit content but also for their implicit influence on behaviour, structures, and decision logic. What decisions are made because of regulation? What decisions are delayed, avoided, or obscured by regulatory complexity? Where do regulatory goals conflict with clinical or ethical imperatives? By answering these questions, high-quality, ethical decision-making can be supported.

In conclusion, in rethinking the healthcare decision-making ecosystem, a critical engagement with regulation, its purpose, performance, and unintended effects, is not optional but foundational. In practical terms, de-engineering is applied as a short, iterative cycle. First, the decision under review is defined, and the criteria for a successful outcome are specified. Second, it is decomposed into its constituent elements, that is, people and roles, rules and criteria, available information, and technologies in use. Third, the connections among these elements are clearly defined. Fourth, risk is identified through an audit of decision thresholds, the origin and quality of the evidence, and fairness. Fifth, a simpler future pathway is assembled, with explicit thresholds, documented exceptions, and human oversight at key points. Sixth, a small set of indicators, such as time to decision, compliance with guidance, and patient-reported experience, is monitored, and adjustments are made based on the findings.

Emerging Technologies

Emerging technologies, particularly artificial intelligence (AI), are rapidly transforming the healthcare decision-making landscape [87]. These technologies are not merely tools for data processing or clinical support, but they are reshaping the very foundations of how decisions are generated, evaluated, and implemented across both clinical and administrative domains [88]. From predictive analytics and machine learning to wearable sensors, decision support systems, and robotic process automation, emerging technologies are challenging traditional roles, workflows, and epistemologies in healthcare [89]. As such, they constitute a vital component of the modern decision-making ecosystem, one that demands careful scrutiny, especially within a de-engineering framework aimed at enhancing transparency, accountability, and resilience.

At the core of this technological transformation is artificial intelligence (AI), which encompasses a range of applications including natural language processing, computer vision, and deep learning algorithms [90]. In clinical settings, AI-powered decision support systems are increasingly used for diagnosis, treatment recommendations, and image interpretation [91]. These systems often outperform human clinicians in narrow, high-volume tasks such as radiographic analysis or ECG interpretation, offering faster, more consistent outputs [92]. When integrated effectively, AI can enhance the accuracy, efficiency, and scalability of clinical decision-making, particularly in resource-limited settings or during public health crises [93]. However, the incorporation of AI into healthcare decisions introduces new forms of complexity [94]. While AI systems operate on statistical correlations and learned patterns from large datasets, they often lack the contextual reasoning, empathy, and ethical discernment that human decision-makers bring. This sociotechnical gap raises questions about accountability, transparency, and interpretability [95]. The "black box" nature of many AI algorithms, especially deep learning models, can make it difficult for clinicians and patients to understand how decisions are generated, undermining trust and shared decision-making. Moreover, biases embedded in training data can lead to unequal or unsafe outcomes. AI models trained on non-representative datasets may inadvertently reproduce or exacerbate existing health disparities, particularly across race, gender, socioeconomic status, or geography [96]. Thus, the decision-making capacity of AI is only as ethical and equitable as the data and logic upon which it is built.

Beyond AI, other emerging technologies are also shaping healthcare decisions. Wearable health technologies, such as smartwatches, biosensors, and mobile health apps, enable real-time monitoring of physiological metrics and lifestyle behaviours. These tools shift some aspects of decision-making from the clinical setting to the patient, supporting models of self-management and personalized care [97]. While empowering, this shift also places new demands on patients' health literacy and data interpretation skills, raising concerns about unequal access to digital health resources and decision fatigue.

Furthermore, telemedicine platforms and remote care technologies have redefined how decisions are made in distributed care environments [98]. Clinicians now make diagnostic and treatment decisions based on data transmitted remotely, sometimes with limited physical examination or patient context. These technologies expand access but also introduce new uncertainties and communication challenges, especially when relying on chatbots for initial decision layers. On the administrative side, technologies like robotic process automation (RPA) and predictive analytics are being used to support resource allocation, patient scheduling, supply chain management, and population health planning [99]. While these tools offer efficiencies, they can depersonalize decisions and introduce new risks if poorly integrated with human oversight. Decisions made based solely on algorithmic outputs may overlook local nuances, staff expertise, or patient preferences.

Within a de-engineering approach, emerging technologies must be examined not only for their functional capabilities but also for their systemic impacts. What decisions are now delegated to machines? What human insights are bypassed or marginalized? How are power dynamics shifting between stakeholders as technology mediates more of the decision-making process? These questions are central to understanding the evolving decision-making ecosystem and ensuring that technological progress aligns with the core goals of healthcare, that is, safety, equity, efficacy, and compassion.

In conclusion, emerging technologies, including but not limited to artificial intelligence, are reshaping the healthcare decision-making ecosystem in profound ways. While they offer significant potential to enhance clinical accuracy, operational efficiency, and patient empowerment, they also introduce new ethical, epistemological, and systemic challenges. As decision-making becomes increasingly digitized, it is essential to interrogate the assumptions, limitations, and consequences of these technologies. A thoughtful, interdisciplinary, and equity-focused approach is required to ensure that technology augments rather than undermines the human dimensions of healthcare decision-making.

Dynamic interplay of decision-making elements

While each element of the healthcare decision-making ecosystem, that is, decision theories, stakeholder roles, leadership styles, organizational cultures, regulatory frameworks, and emerging technologies, can be examined in isolation, their true influence emerges from the dynamic interplay among them. It is this multi-layered, often nonlinear, and sometimes contradictory interaction that gives rise to the complexity and unpredictability that characterizes real-world healthcare decision-making. These interdependencies are not merely additive; they are synergistic and sometimes antagonistic, producing emergent behaviours that cannot be predicted solely by analyzing individual components. In practice, whether such interactions lead to coherence or breakdown is shaped by system-level enablers, that is, leadership commitment, organizational culture, access to high-quality evidence, continuous professional development, meaningful stakeholder engagement, and fit-for-purpose digital solutions, rather than by any single component alone.

For instance, a well-designed clinical decision support system (technology) may rely on normative decision models and evidence-based guidelines (decision theory), but its effectiveness depends heavily on organizational culture (openness to innovation), leadership paradigms (support for change management), and regulatory compliance (data privacy, documentation). If frontline stakeholders do not trust the system or feel it undermines their professional autonomy, adoption may fail, even if the tool is technologically sound. Similarly, a shared decision-making approach rooted in phenomenological reasoning may be ethically ideal but can be undermined by time pressures, hierarchical culture, or misaligned reimbursement structures.

These cross-cutting influences create tensions and feedback loops. Cross-cutting influences generate (a) value tensions related to safety and efficiency, (b) temporal tensions including short-term targets and long-term outcomes, and (c) jurisdictional tensions concerning clinical autonomy and managerial oversight. These tensions manifest through feedback loops. Mapping these loops reveals bottlenecks, latent contradictions, and changing points that are not evident in linear analysis. Regulations meant to standardize care may unintentionally suppress clinical intuition or context-specific judgment. Leadership styles that promote rapid innovation may clash with cautious regulatory bodies or entrenched professional norms. Technological tools that streamline administrative processes may inadvertently reinforce inequities if they exclude certain populations or overlook stakeholder values. In this sense, decision-making complexity arises not from too many variables, but from conflicting but simultaneously interwined different parts of the ecosystem.

This complexity is further amplified by uncertainty and unpredictability inherent in the healthcare ecosystem. Patient responses to treatment are not foreseen in advance. Public health crises evolve rapidly. Evidence-based shift as new research emerges. In such conditions, rigid decision frameworks are often insufficient, and decision-makers must improvise using partial information, guided by experience, intuition, and contextual reasoning. The interplay of structured systems (e.g., guidelines, algorithms) and human elements (e.g., empathy, moral reasoning) adds further layers of unpredictability.

Within this context, the concept of de-engineering becomes especially relevant. Traditional systems design assumes that complex systems can be optimized by breaking them down into simpler, controllable parts [100]. However, in healthcare, the interactions between these parts generate complexity that cannot be managed solely through control mechanisms. De-engineering, therefore, offers a complementary approach that seeks to dissect, challenge, and reveal the hidden interactions, misalignments, and contradictions embedded within the ecosystem. It asks not only "What does each part do?" but also "How do parts interact, interfere, or reinforce one another, and to what effect?".

By mapping these interdependencies, de-engineering enables a more nuanced understanding of where decision-making bottlenecks occur, how authority is distributed or concentrated, and why some decisions succeed while others fail. For example, uncovering how organizational silos prevent cross-functional decision-making can inform structural redesign. Identifying how misaligned incentives distort clinical judgment can guide policy reform. Recognizing where AI tools replace rather than augment human reasoning can prompt a recalibration of technology integration.

In sum, the complexity and unpredictability of healthcare decision-making are not the result of flawed individuals or broken parts, but of deeply entangled systems where elements interact in dynamic and often opaque ways. Through the lens of de-engineering, we gain the conceptual and analytical tools to unravel these interactions, diagnose dysfunction, and design interventions that honour both the systemic nature of decision-making and the human values it ultimately serves.

## Conclusions

Healthcare decision-making is not governed by a single actor but by a stack of mutually reinforcing entities. The proposed de-engineering approach focuses on identifying decision points embedded in each micro-element of the healthcare decision-making macro-system by examining their strengths and limitations. By decomposing layered structures and making their interrelations explicit, the healthcare ecosystem can deliver decisions that are not only accurate but also explainable and resilient. This paper reframed healthcare decision-making from an ecosystem perspective and advanced de-engineering as a tool for making healthcare decision-making easily understandable. Indeed, rather than treating decisions as isolated choices or purely technical optimizations, de-engineering decomposes the healthcare decision-making into its constituent elements, such as decision theories and reasoning modes, stakeholders’ qualities and roles, leadership paradigms, organizational cultures, regulatory frameworks, and emerging technologies. This work is primarily conceptual and limited by the absence of empirical validation.
